# Prolonged GnRH suppression period in controlled ovarian hyperstimulation cycles: Impacts on IVF outcomes?

**Published:** 2012-03

**Authors:** Ozlem Gun Eryilmaz, Esma Sarikaya, Melike Doganay, Leyla Mollamahmutoglu, Nedim Cicek

**Keywords:** *GnRH agonist*, *IVF outcome*, *Embryo quality*

## Abstract

**Background: **Prolonged GnRH-a administration in IVF cycles may have some advantages related to the treatment outcomes.

**Objective:** In this study, we aimed to analyse the effect of prolonged gonadotropin releasing hormone agonist (GnRH-a) administration on controlled ovarian hyperstimulation outcomes of in vitro fertilization (IVF) patients.

**Materials and Methods:** In this retrospective study, 55 patients with a GnRH-a administration period more than 10 days were compared with 55 patients whose same period was ≤10 days with respect to the demographic characteristics, metaphase II (MII) oocyte ratio, grade I (GI) embryo ratio, blastocyst ratio, fertilization, implantation, and the clinical pregnancy rates.

**Results:** The mean hospital visit count of the prolonged GnRH-a patients was 2.6±0.4. As we expected, total GnRH-a doses used during hypophyseal down regulation were significantly different between the groups (p<0.0001). MII oocyte, G1 embryo and the blastocyst ratios were also significantly different between the groups (p<0.0001; p<0.01 and p<0.05). All the other parameters were insignificant.

**Conclusion:** Prolonged GnRH-a administration during ovarian suppression in IVF patients may have positive impacts on the oocytes and the embryos, but this affect may not be observed in the overall pregnancy rates.

## Introduction

Gonadotropin releasing hormone (GnRH) is the primary hypothalamic regulator of reproductive function. Synthetic GnRH causes a huge follicle stimulating hormone (FSH) and luteinizing hormone (LH) release from the pituitary gland and this agonistic activity was the reason why they were called as GnRH agonist (GnRH-a). 

It has been used in in-vitro fertilization (IVF) programs since the 1980s, and the main advantages were related with lesser cancellation rate, prevention of premature LH surge (1), and higher oocyte recruitment in poor responder patients (2). The optimal dose and the duration of GnRH-a administration in hypophyseal suppression is not clear (3). 

Jahnsens conducted a dose finding study and declared the needed dose to prevent LH surge was less than the doses needed for malignant diseases (4). Recent studies were mostly concentrated on the optimal beginning time of the GnRH-a; follicular, early luteal, or late luteal (5) and the effects on the endometrial cells. 

Loutradis compared the IVF outcomes of prolonged GnRH-a in a small group of patients whose administration period was less and more than 15 days, and found the favourable effects of GnRH-a prolongation on embryo cleavage speed and pregnancy rate (6). There is no other similar study in the literature related to the chronology. During controlled ovarian hyperstimulation (COH) programs, some patients reach the adequate hormonal profile of down regulation with E2 <50 pg/ml (7) in a few days like 7 to 10 days, and in some patients this time is more than 10 days. 

In this study, we compared the IVF outcomes of the patients whose adequate ovarian suppression period was less than 10 days versus more than 10 days. 

## Materials and methods

This retrospective, single-institution, cross-sectional analysis was conducted between January-December 2009 in Zekai Tahir Burak Women’s Training and Research Hospital, IVF Department, Ankara, Turkey. 

During this period, number of the patients who were treated with long agonist IVF protocol was 489, and among these, patients with a GnRH-a suppression period >10 days (Group A, n=55) were compared with the patients with a GnRH-a suppression period ≤10 days (Group B, n=55) with respect to age, body mass index (BMI), hospital visit count, preantral follicle count, duration and cause of infertility, basal E_2_, FSH, and LH levels, total doses and the duration of GnRH-a administration during the hypophyseal down regulation period, duration of induction, total gonadotropin doses used, follicle count >16mm, peak E_2_ level and endometrial thickness on hCG day, number of aspirated oocytes, metaphase II (MII) oocyte ratio, grade I (GI) embryo ratio, blastocyst ratio, fertilization, implantation, and the clinical pregnancy rates. 

Patients, who were at the primary infertility age, 20-38 years, were included. Patients with FSH>13 Mıu/ml were excluded from the study. In no instance was donor sperm or oocyte used for ICSI since it is forbidden by law in Turkey. This study was approved by the local Ethics Committee.

**Study design**

Leuprolide acetate (Lucrin, Abbott, Istanbul) was started in the mid lutel phase of the cycle at a dose of 0.5mg/day subcutaneously (SC). Menstrual bleeding was the sign of the adequate ovarian suppression during GnRH-a administration which was confirmed with the serum levels of E_2_<50 pg/mL and LH<5 IU/mL without any ovarian mass (7). The comparison group had confirmed the suppression in the period of 10 days, and this was assessed via the beginning of the menstrual bleeding with the suppressed hormonal levels or it was stated with only the adequate hormonal results in the patients without menstrual bleeding which was measured at the 10^th^ day. 

If the ovarian suppression was not achieved, GnRH-a dose was increased, and the patient was called back in 6 to 7 days later for repeat E_2_ and LH measurements. Increasing the dose and the duration of the GnRH-a had continued in a period of about 3 weeks, and they set up the study group. After achievement of the adequate hypophyseal suppression recombinant FSH stimulation was initiated and at that time, the dose of leuprolide acetate was decreased to 0.25 mg/day. Further recombinant FSH doses were determined according to the standard criterion for follicular maturation assessed by ultrasound and serum E_2_ measurements.

250 µg recombinant hCG (r-hCG) (Ovitrelle, Merck Serono, Italy) was administered when at least three follicles had reached a diameter of 18 mm. Transvaginal-guided oocyte retrieval was done under general anesthesia 36 hours after the hCG injection. The morphological grading of the oocytes was done according to oocyte-cumulus complex, and embryo transfer was done between the 2^nd^ to 5^th^ days.

**Statistical analysis**

The data was analysed with SPSS 11.0 package program. The observed power computed using α=0.05 was 0.80 for the present study. Independent sample’s t-test, Mann-Whitney U test, and Pearson-chi square test were used for the analysis. p<0.05 was accepted as significant.

## Results

Demographic variables of the patients are summarized in table I. Infertility reasons were grouped as unexplained, endometriosis, male, tubal, and ovulatory factors. Age, infertility reasons and the durations were insignificant between the groups. The mean hospital visit count of the prolonged GnRH-a patients was 2.6±0.4. The minimum and the maximum durations of the cycles in the patients of prolonged GnRH-a group were 22 and 39 days; respectively. Hormonal profile and the IVF outcomes are shown in table II. 

As we expected total GnRH-a doses used during hypophyseal down regulation were significantly different between the groups (p<0.0001). MII oocyte ratio of the patients with GnRH-a suppression period less than 10 days was significantly different from the patients whose suppression period was more than 10 days (p<0.0001). 

Grade I embryo and the blastocyst ratios were significantly more in the patients of GnRHa suppression >10 days (p<0.01 and p<0.05 respectively), but clinical pregnancy rates were similar.

**Table I:** Demographic data of the patients of GnRH-a administration period ≤10 days and >10 days

		**Period ≤ 10 days (n=55)**	**Period > 10 days (n=55)**	**p-value**
Age (years)		28.9 ± 4	29.3 ± 3.8	0.675
Infertility duration (years)		8.1 ± 4.3	8.5 ± 3.4	0.282
Infertility causes				
	Unexplained	21/55 (39.2 %)	20/55 (37.0 %)	0.841
	Ovulatory	10/55 (18.5 %)	12/55 (22.2 %)	0.841
	Endometriosis	7/55 (13.1 %)	6/55 (11.2 %)	0.764
	Male	14/55 (25.5 %)	17/55 (29.6 %)	0.823
	Tubal	3/55 (3.7 %)	0/55 (0.0 %)	0.241
	Hospital visit count	1.0 ± 0.0	2.6 ± 0.4	0.0001

Values are expressed as mean±SD. NS: Not significant.

**Table II:** Cycle characteristics of the patients of GnRH-a administration period ≤ 10 days and > 10 days

	**Period ≤ 10 days (n=55)**	**Period > 10 days (n=55)**	**p-value**
BMI¹	26.5 ±4.0	25.2 ±4.4	0.189
PCO² ratio	22/55 (40.7 %)	19/55 (35.1 %)	0.899
Basal E2 levels(pg/ml)	47.9 ±15.7	43.2 ±19.9	0.289
D3 FSH(mIU/ml)	8.6 ±1.2	6.9 ±1.5	0.020
D3 LH(mIU/ml)	5.0 ±3.1	5.6 ±2.7	0.327
GnRHa dose³(IU)	118.3 ±16.3	230 ±46.3	<0.0001
Total gonadotropin used(IU)	1793.3 ±695	2240.9 ±1563.4	0.599
Stimulation duration(days)	10.5 ±1.8	10.7 ±2.0	0.547
HCG E2	1960.1 ±960.6	1995.1 ±1085.6	0.886
HCG endometrium	10.2 ±1.5	10.5 ±2.0	0.462
No. of follicles≥16mm	5.5 ±3.2	5.3 ±2.8	0.734
No. of oocytes retrieved	8.5 ±4.8	8.7 ±5.8	0.831
No. of MII oocytes	6.1 ±4.0	6.9 ±5.1	0.457
MII oocyte ratio	338/489 (69.3 %)	351/426 (82.5 %)	<0.0001
Fertilization rate	242/489 (49.5 %)	222/426 (52.2 %)	0.466
No. of embryos transfered	1.7 ±1.0	2.3 ±0.8	0.800
Grade I embryos ratio	16/120 (13.6 %)	36/138 (26.2 %)	<0.01
Blastocyst ratio	9/120 (8.1 %)	28/138 (20.5 %)	<0.05
Implantation rate	27/120 (22.9 %)	23/138 (17 %)	0.305
Clinical Pregnancy rate	22/120 (18.7 %)	17/138 (12.8 %)	0.241

Values are expressed as mean ± SD. NS: Not significant.

¹ Body mass index;

² Polycystic ovary;

³ Dose during suppression period.

## Discussion

GnRH-a treatment is used for hypophyseal down regulation in long protocols of IVF treatments and adequate hypophyseal suppression was achieved when E_2_ and LH levels were less than 50 pg/mL and 5 IU/mL respectively. Hormonal levels of ovarian suppression were reached in almost three weeks of administration (3). In this study, COH outcome differences were analysed with the acceptance of the 10days as the point of act. 

GnRH-a prolongation for adequate ovarian suppression needed much more GnRH-a doses. Loutradis *et al* (6) had found a favorable status of GnRH-a prolongation in embryo cleavage speed and pregnancy rates. In his study, the compared durations were less and more than 15 days. In our study, 10 day was the period of patient selection, and the results of prolonged suppression supported Loutradis’s study. Patients with a prolonged suppression duration more than 10 days had more qualified embryos with a significantly more G1, and blastocyst formation ratio.

GnRH-a effect on endometrial cells was studied many times and the results were variable. Meresman *et al* had concluded the apoptotic effect of GnRH-a on the endometrium (8). In this study, the more doses of GnRH-a usage was not related with any kind of endometrial disturbance. Endometrial measurements of the patients with prolonged GnRH-a usage was comparable with the others on ultrasonographic appearances. 

This conclusion was supported with our results, that the endometrial thickness on the hCG day was comparable between the groups. Endometrial matrix proteins and their inhibitors was studied by Chou *et al* who found that GnRH can modulate the cyclic remodelling events before implantation (9). The promoter effect of GnRH-a on embryo development and implantation was also reported (10, 11). 

Kawamura *et al* documented the antiapoptotic effect on mouse blastocysts which was parallel with our results with a significantly more blastocyst formation in prolonged GnRH-a used IVF cycles (10). In a recent study by Klemmt *et al*, the effect on embryo invasion was analysed, and no negative impact was found (12). In our study, GnRH-a prolongation with higher doses did improve the oocyte and the embryo, but not the implantation and the clinical pregnancy rates. 

This may be because of the apoptotic effect of the prolonged GnRH administration on the endometrium that the implantation was not improved even with a qualified embryo. This apoptotic effect was not shown in the gross thickness measurements of the endometrium, but undetectable biochemicals that may be released into the microenvironment could negatively impact the implantation process itself. GnRH-a may be successful in the role of the ovarian suppression, and in the ovarian improvement with qualified oocytes, but it may cause some negative effects on the endometrium that implantation rates may not be affected in the same positive manner. 

It is known that age more than 35 years was a poor prognostic factor for IVF success (13), but this poor expectation was not seen in the prolonged GnRH-a suppression period. The insignificant age correlation showed that older patients did not have a risk of suppression failure in long agonist protocols. Increased BMI also was not a predictive criterion for prolonged GnRH-a administration need. Patients with larger fatty mass in their bodies did not have an unfavorable hormonal status during hypophyseal down regulation. 

Their gonadotropin initiation time was comparable with the lean patients. Adequate ovarian suppression in 10 days did not show any specificity from the ovarian reserve point of view. Ovaries with a high number of follicle count, like in polycystic appeared ovaries were not different from the other lower follicle counted ovaries in GnRH-a suppression duration and doses. An ovary containing lots of prenatal follicles did not needed higher GnRH-a doses, and the expectation of a prolonged suppression period was not reliable. The increased hospital visit count in patients of prolonged suppression period was the only negative side. Prolongation caused almost three times more hospital attendance in these patients. 

In summary, prolonged GnRH-a administration during ovarian suppression may have positive impacts on the oocytes and the embryos. But this effect was not observed in the overall pregnancy rates. Long suppression period with more hospital visits may tire the patients, but it may be a chance to achieve more qualified oocytes and embryos. 
